# The preliminary measurement properties of the person-centred community care inventory (PERCCI)

**DOI:** 10.1007/s11136-018-1917-1

**Published:** 2018-06-19

**Authors:** Mark Wilberforce, David Challis, Linda Davies, Michael P. Kelly, Chris Roberts

**Affiliations:** 1 Social Policy Research Unit, Department of Social Policy and Social Work, University of York, York, UK; 2 Personal Social Services Research Unit, School of Health Sciences, University of Manchester, Manchester, UK; 3 Manchester Centre for Health Economics, School of Health Sciences, University of Manchester, Manchester, UK; 4 Institute of Public Health, University of Cambridge, Cambridge, UK; 5 Centre for Biostatistics, School of Health Sciences, University of Manchester, Manchester, UK

**Keywords:** Person-centred care, Patient-centred medicine, Older people, Dementia, Community care, Social care, Psychometrics, Patient experience, Measurement

## Abstract

**Purpose:**

Researchers investigating person-centredness in older people’s long-term community care are hindered by the lack of appropriate measures. Studies have tended to rely on proxy indicators or generic instruments, risking invalid results. This new research aimed to develop and psychometrically test a person-centredness scale for use in older people’s community services.

**Methods:**

Questionnaire items were sourced from groups of older people and mapped to a conceptual framework of person-centredness. A postal questionnaire in 2015–2016 tested these items with older people supported by mental health and social care services in five areas of England. Dimensionality was assessed through exploratory factor analysis and a confirmatory bifactor model, with classical item analysis removing weak items. Test–retest analysis was undertaken through a repeated postal questionnaire 3 weeks after the first.

**Results:**

Three factors were identified, representing (i) interpersonal and (ii) organisational aspects of person-centred care; and (iii) negatively phrased items. Removing weaker items resulted in an 18-item scale. The bifactor analysis concluded the summary scale was ‘essentially unidimensional’. The Person-centred community care inventory (PERCCI) had excellent reliability, with Intra-Class Correlation Coefficient of 0.886 [95% CI 0.818–0.929]. A priori hypotheses about associations with satisfaction metrics and support variables were broadly confirmed.

**Conclusions:**

The PERCCI has promising measurement properties and can be recommended for use in research with older adults using community mental health and social care services. Future developments must identify how sensitive the instrument is in detecting changing service quality.

## Introduction

The language of ‘person-centredness’ is ubiquitous and forms the central plank of most quality improvement strategies across health and care systems worldwide [1]. Although the term’s precise meaning varies between service settings, it can generally be understood to encompass approaches to care provision that recognises, respects and responds to the uniqueness of each individual [2]. The term is commonly used as a critique of approaches to health care delivery which privilege biomedical understanding of disease [3, 4], on the grounds that these can leave important needs unaddressed [5]. The influence of person-centredness can scarcely be overstated. The World Health Organization recently proposed a “fundamental paradigm shift” in service design based on person-centred principles [1], and has called for a “re-examination of medicine and health care to refocus the field on genuinely person-centered care” [6]. However, a near-unanimous view is held that the quality of evidence underpinning person-centredness fails to match its high status in policy rhetoric [7–9]. Crucially, measurement problems have been identified as a leading contributor to inconclusive and low-quality evidence [10–13], and in a review of expert opinion has been identified as a priority theme for action [14].

Particular measurement challenges exist in community services for older people with long-term care needs [3]. Two difficulties stand out. First, person-centredness in the context of later life care has a distinctly different emphasis to that promulgated in mainstream policy [2], which is not reflected in many extant instruments [15]. For example, authors in gerontological nursing have noted a ‘youthful bias’ in common interpretations of person-centredness [16], with its focus on individuality, self-determination, autonomy and choice in care. In the context of later life services for people with memory and mental health problems, person-centred approaches instead place an emphasis on the interpersonal interaction and interdependencies involved in care [17], and how these can be used to reinforce personhood [18]. New measures for these settings should thus address the priorities of older people themselves. Second, whilst some instruments have been designed for older people’s care, they have almost exclusively been used in residential and institutional settings, often based on resource-intensive observation of care interactions [12]. Two recent systematic reviews highlighted the absence of measurement tools suitable for home- and community-based care suggesting that these are important research areas [3, 19].

The absence of high-quality measures have important consequences. Some researchers have turned to proxy instruments such as simple satisfaction ratings, which have been criticised as being unsuited to the task [3, 14, 20]. For example, equivocal results in an RCT evaluating a person-centred intervention concluded that broad experience metrics were insufficiently sensitive to identify meaningful change [21]. Elsewhere, Edvardsson and Innes [12] lamented the lack of direct measures of person-centredness in dementia care trials, noting a tendency to use prescription rates of neuroleptics as a proxy for whether care approaches were more or less person-centred. Researchers have been encouraged to “move examinations away from structural proxies …and towards more meaningful measures” [20].

This new study aimed to design and psychometrically test a new measure of person-centredness to evaluate older people’s experiences of community mental health and social care.

## Methods

The study comprised the design and implementation of a preliminary 30-item postal questionnaire for self-completion by service users and their families, which through psychometric testing was reduced to a shorter scale with optimised measurement properties.

### Phase 1: item development and pre-testing

The first stage sought to establish a pool of potential questionnaire items. (Details of this phase have been reported elsewhere [22]. The method used is outlined here to assist in understanding the results of subsequent psychometric testing.) To this end, two groups of older people were recruited through voluntary sector providers of mental health services in the North West of England, one serving a predominantly white population and another for those of south Asian heritage. These 39 participants were asked to brainstorm statements that described a good or bad care experience, following a concept mapping methodology [23] increasingly used for questionnaire development [24]. The two groups generated 131 statements. The study’s patient and carer advisory group suggested two separate classes of questions could be incorporated into the design: those describing interpersonal quality (between user and care worker), and those describing organisational features (between user and agency/provider).

To support content validity, each statement was then mapped to a literature-based concept framework of person-centredness specifically developed for this purpose (and published elsewhere [2]). That framework identified 12 attributes of person-centredness under three key themes: (a) understanding the person; (b) promoting the care relationship; and (c) engagement in decision-making. Only those statements that could be justified as being an articulation of one of these person-centred attributes were retained. Statements that were semantically equivalent were also removed leaving 59 suitable candidate items.

The statements were reformulated as Likert items for use in a self-completed questionnaire. The questionnaire was ‘pre-tested’ through think-aloud and cognitive debriefing methods [25] with 14 older people (eight in tandem with another family members). Items that did not work well were either reformulated or replaced with an alternative statement mapped to the same component of person-centredness. This testing also led to a reduction in the number of response options from five to four. A final instrument for wider psychometric testing comprised 30 items on a four-point Likert scale.

### Phase 2: psychometric testing

A postal survey was undertaken between October 2015 and May 2016 to provide quantitative data for psychometric testing.

#### Participants and settings

Participants were home-dwelling service users on the active caseloads of integrated community mental health and social care services for older people [26], excluding (i) those without capacity to consent; (ii) those with moderate to severe dementia and/or (iii) in crisis or hospital.

The research was undertaken within the catchment of five English NHS Mental Health Trusts. In four Trusts, delivery of questionnaires was organised through a central mailing with a second ‘reminder’ questionnaire sent to non-respondents after 2 weeks. The Trust supplied matched administrative data capturing information on age, gender, broad diagnostic group, date of referral and service receipt, and provided summary data for non-respondents. In addition, a test–retest questionnaire was administered to early responders until the target sample size (below) was achieved. The fifth Trust was not sufficiently resourced to administer a central mailing, and so questionnaires were hand-delivered through the care coordinator. No reminder questionnaire, test–retest questionnaire or matched administrative data were undertaken in this Trust. In all five Trusts, the questionnaire was returned by respondents in a sealed freepost envelope direct to the research team.

#### Sample size

There are no definitive a priori calculations to support sample size choices for psychometric testing [27]. A crude rule-of-thumb expects a minimum of 10 responses per questionnaire item (*n* = 300), but to account for missing values and uncertain response rates, larger recruitment efforts were indicated. The research team managed these risks by aiming to distribute 2000 questionnaires to achieve a sample in excess of 300. For test–retest inspection, a minimum sample of 50 was sought [27].

#### Psychometric analysis

Dimensionality was assessed initially through exploratory factor analysis (EFA). It is known that factor analysis with Likert-type items tend to over-factorise using the eigenvalue > 1 rule for factor retention [28]. Parallel analysis [29] using polychoric correlations was therefore undertaken, using FACTOR software with missing data addressed using the (in-built) hot-deck multiple imputation procedures from five imputed datasets [30]. A factor was only retained if its eigenvalue exceeded its counterpart obtained from randomly generated data. An oblique rotation allowed for correlated factors.

The researchers then sought to reduce the item set to improve its efficiency using widely applied metrics to identify potential weakness (see Box 1). Whilst the literature provide some decision rules for choosing items [31], modern guidance suggests these should not be applied mechanically [32], with greater weight attached to the value of the item’s wording/content. Transparency in this process is regarded as crucial [33] and is bolstered here by describing the rationale for item exclusion as part of the findings below.

**Box 1:** Framework for identifying potential weak items

Dimensionality	Low loadings (e.g. < 0.4) or high loadings on > 1 factor
Internal consistency	Low item-total correlations (e.g. < 0.5)
Reliability	Low weighted kappa statistic (e.g. < 0.50) from test–retest analysis
Floor/ceiling	Large proportion of sample at extreme value (e.g. > 2/3)
Missing items	Large proportion of sample missing item (e.g. > 10%)

Moving to the shortened scale, the factor structure was re-inspected for the retained items as a Confirmatory Factor Analysis using a weighted least squares approach in MPlus. This analysis compared a three-factor solution suggested by the initial EFA with a bifactor model [34]. Bifactor models are used in situations in which multiple factors are highly correlated, often with dominant first factor eigenvalues, indicating the presence of a ‘general factor’ in addition to separate subscales. This provides evidence supporting the use of a single summary score from items forming the individual subscales. Two statistics were used to evaluate the explanatory power of a general factor in a bifactor representation: the Explained Common Variance (ECV) and OmegaH [35]. The ECV is calculated as the ratio of variance explained by the general factor to the variance explained by all factors combined; an ECV > 0.7 is a suggested threshold to determine that common variance is ‘essentially unidimensional’ [36]. OmegaH is the proportion of total variance in the model attributable to differences in the general factor; a threshold of omegaH > 0.8 has been proposed [36].

Test–retest analysis was in the form of kappa statistic for individual items (squared weighting) and an Intra-Class Correlation Coefficient (two-way random-effects model) and Bland–Altman Limits of Agreement for scale reliability. Further, in the absence of any suitable measure of person-centredness for use in the community, a preliminary and pragmatic assessment of criterion-related validity was provided by correlation with contemporaneously collected satisfaction score and the Friends and Family Test (both with 5-point Likert response options). A moderate positive correlation was anticipated. An exploratory regression was also undertaken to examine associations with collected variables. Literature-informed a priori hypotheses were that respondents receiving help from community mental health support workers would report higher scores of person-centredness [37], whilst those receiving domiciliary care would report lower scores. These analyses were conducted in Stata [38]. A final exploration repeated the analyses above but removing negatively phrased items to show the psychometric implications of doing so.

The research received ethics permission from Greater Manchester South NRES ethics committee (Ref: 14/NW/0303).

## Findings

Of 612 returned questionnaires, 16 were excluded because no data could be entered, being blank, consisting of only written accounts in the margins rather than completed schedules, or providing only ineligible responses (having ticked multiple boxes for the same item). Data from 596 usable questionnaires were analysed, representing a final response rate of 29%. Respondent characteristics are presented in Table 1, with key groups being well represented except for the oldest age-group (> 90 years) with only 14 completed questionnaires. When compared with non-respondents—using administrative data available in only four Trusts (see above)—respondents were only marginally younger on average (75.3 vs. 76.9 years old). However, respondents were less likely than non-respondents to be supported with an organic illness (31.4 vs. 42.2%) and to have spent longer than 2 years on the team caseload (28.5% vs. 15.1%). Questionnaires were generally well completed with just 3% of items being unanswered, and with 96% of schedules having at most five missing items. The likelihood of item non-response increased towards later items in the schedule, perhaps indicative of fatigue. Returning an incomplete questionnaire was positively related to age group (*χ*^2^(3) = 11.38, *p* = .010) but to no other variable.

**Table 1:** Respondent characteristics

	Respondents (*n*)	Respondents (%)
Gender (missing = 38)		
Female	344	61.7
Male	214	38.4
Age (missing = 15)		
Under 70	156	26.9
70–79	280	48.2
80–89	131	22.6
90 or over	14	2.4
Diagnosis^a^ (missing = 53)		
Non-psychotic functional disorders	189	44.8
Psychosis	101	23.9
Organic	132	31.3
Service receipt (missing = 38)		
Receiving homecare	163	27.6
Length of time on caseload^a^ (missing = 37)		
Less than 6 months	81	18.5
Between 6 and 12 months	121	27.6
Between 12 and 18 months	69	15.8
Between 18 and 24 months	42	9.6
Over 2 years	125	28.5

^a^Available from matched administrative data for four of the five Trusts involved

### Exploratory factor analysis

Exploratory Factor Analysis indicated a dominant first factor (eigenvalue 16.3) accounting for 54% of variance, with three additional factors having eigenvalues > 1.0 (k = 1.9, 1.8, 1.1) together explaining a further 12%. Parallel analysis recommended the retention of only the first three factors. Under an oblique rotation, the loading patterns for the three retained factors are presented in Table 2. Factors 1 and 2 represented positively phrased items relating to interpersonal and organisational aspects of person-centred care, respectively. The third factor represented the negatively phrased items.

**Table 2:** Exploratory factor analysis—rotated (geomin) factor loadings

	Factor 1	Factor 2	Factor 3
Q1	0.815		
Q2	0.856		
Q3	0.777		
Q4	0.508		
Q5^a^			0.319
Q6	1.038		
Q7	1.047		
Q8	0.904		
Q9	0.839		
Q10	0.869		
Q11^a^			0.341
Q12	0.686		
Q13	0.850		
Q14	0.771		
Q15	0.804		
Q16^a^			0.422
Q17		0.666	
Q18		0.857	
Q19		0.821	
Q20^a^			0.463
Q21^a^			0.494
Q22		0.476	
Q23^a^			0.721
Q24^a^			0.587
Q25		0.772	
Q26		0.894	
Q27		0.904	
Q28		0.818	
Q29		0.730	
Q30^a^			0.574
Eigenvalue	16.39	1.89	1.80
Variance explained	54.6%	60.9%	66.9%

^a^Reverse scored. Loadings < 0.30 suppressed

### Item reduction

Table 3 presents information used to support item reduction for each scale. Four items were removed in relation to Factor 1. Q3 had relatively weak reliability whilst Q4 had nearly three-quarters of the sample at the ceiling. Q12 was removed since both reliability and ceiling issues were identified, and several other items already captured the ‘respectful interactions’ component of person-centredness. Finally, Q2 was removed since two other items—as shown in Table 3—already captured this component of person-centredness, and the authors felt the content of the other two items was preferable (referring to the pre-testing of the instrument), and they had fewer respondents at the ceiling.

**Table 3:** Summary information for item-level analysis and scale reduction

Item	Component of person-centredness	Item-total correlation			Reliability (kappa)	% at max	% missing	Retention decision
		Factor 1	Factor 2	Factor 3				
Q1	Personal identity	0.818			0.69	67	2.1	✓
Q2	Personal experience of illness	0.832			0.64	56	2.5	×
Q3	Respectful interactions	0.834			0.48	67	0.8	×
Q4	Reciprocity in relationships	0.809			0.57	74	0.5	×
Q5^a^	Tailored care			0.564	0.38	65	2.5	×
Q6	Personal experience of illness	0.790			0.61	44	1.6	✓
Q7	Personal experience of illness	0.783			0.61	41	1.7	✓
Q8	Reciprocity in relationships	0.842			0.69	51	1.3	✓
Q9	Respectful interactions	0.839			0.64	68	1.0	✓
Q10	Personal identity	0.847			0.66	51	1.7	✓
Q11^a^	Respectful interactions			0.562	0.36	86	1.4	×
Q12	Respectful interactions	0.725			0.50	70	2.8	×
Q13	Dimensions needing support	0.820			0.60	59	1.7	✓
Q14	Respectful interactions	0.810			0.66	59	1.6	✓
Q15	Respectful interactions	0.735			0.53	80	1.0	×
Q16^a^	Respectful interactions			0.576	0.57	53	1.6	✓
Q17	Involved in decisions		0.751		0.52	30	2.2	✓
Q18	Positive attitude to capabilities and roles		0.768		0.46	20	7.7	×
Q19	Tailored care		0.830		0.57	29	4.3	✓
Q20^a^	Tailored care			0.595	0.36	23	5.1	×
Q21^a^	Continuity of care			0.632	0.50	37	4.0	✓
Q22	Tailored care		0.694		0.37	33	2.1	×
Q23^a^	Positive attitude to capabilities and roles			0.629	0.56	21	6.3	×
Q24^a^	Involved in decisions			0.541	0.55	24	5.7	×
Q25	Involved in decisions		0.724		0.52	34	3.0	✓
Q26	Involved in decisions		0.744		0.62	36	2.7	✓
Q27	Positive attitude to capabilities and roles		0.754		0.61	30	4.0	✓
Q28	Positive attitude to capabilities and roles		0.692		0.65	23	6.0	✓
Q29	Positive attitude to capabilities and roles		0.795		0.63	24	4.1	✓
Q30^a^	Involved in decisions			0.644	0.59	22	5.4	✓

^a^Reverse-scored items

In relation to Factor 2, two items were removed. Q18 had poor reliability and a large proportion of missing items, potentially because not all people would necessarily want or need to be ‘kept in touch with the local community’. Q22 had low reliability, which, since it was based on whether appointments were kept, may indicate that the item was very sensitive to the most recent experience. Finally, in relation to Factor 3, five items were removed. Q5, Q11, Q20 and Q22 had very poor reliability (κ < 0.4) and their retention could not be justified. Q24 and Q30 both related to involvement in decisions, which were well represented in Factor 2, and so only the latter was retained as having better item-total correlation and fewer missing values. The final 18 items forming the person-centred community care inventory (PERCCI) are presented in Box 2.

**Box 2:** 18 items forming the three subscales of the PERCCI

Interpersonal aspects Alpha = 0.935	Q1: They show an interest in me as a person
	Q6: They know me well enough to recognize when I’m feeling down
	Q7: They can tell my good days from my bad days
	Q8: I have developed a close connection with them
	Q9: They are genuinely caring, not just going through the motions
	Q10: They really understand me
	Q13: They understand the areas of my life that I need help with
	Q14: I am given enough time to say everything that I want to say
	Q15: They speak to me in a friendly and respectful manner
Service aspects Alpha = 0.901	Q17: I have a say in decisions taken about my care and support
	Q19: I get help with things that are most important to me
	Q25: My opinions about my care and support are respected
	Q26: They are interested in my views about my care and support
	Q27: My care and support helps me to feel optimistic about what I can still do
	Q28: I am given opportunity to join groups where I can meet other people
	Q29: My care and support helps me to build confidence
Reverse-scored items Alpha = 0.501	Q16: I feel that I must do as I’m told
	Q21: I see too many different staff
	Q30: Services are too focused on the paperwork, rather than the care

Question numbers relate to their place in the original 30-item questionnaire tested in this paper

### Summary score: a bifactor model

Having established three viable subscales from 18 items, attention turned to the potential for calculating a summary score representing a common ‘person-centredness’ factor. Evidence from the exploratory factor analysis (above) implied that the data structure may be characterised by a single common factor. For example, the ratio of first to second eigenvalue (= 8.6) was very large, and the first and second factors were strongly correlated (*r* = .759). A bifactor model was therefore estimated for the 18 items and compared against the 3-factor solution identified above.

**Fig. 1:**
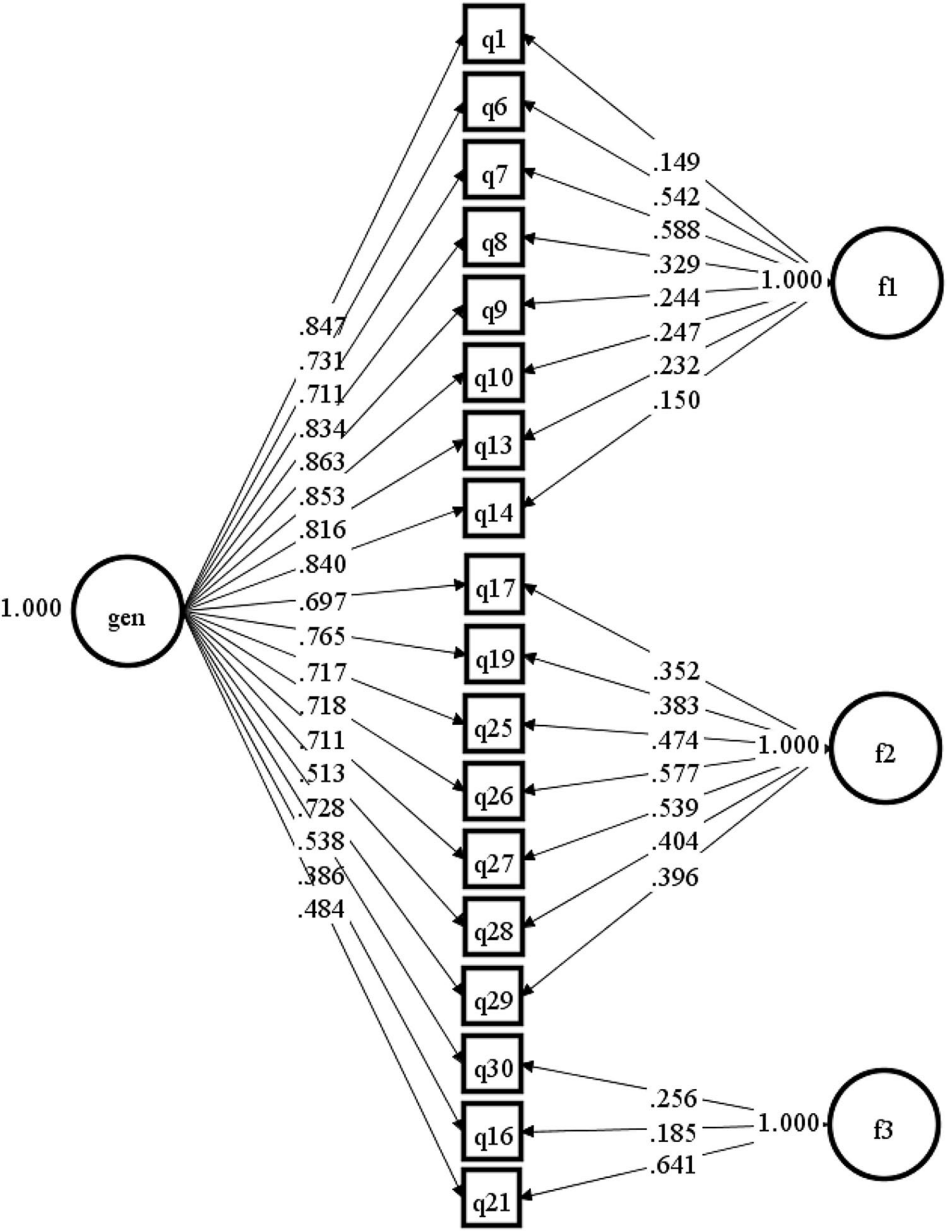
Bifactor model results on 18 items

The results of model fit (Table 4) indicated that the bifactor model was a good representation of the data (although fit indices do tend to favour bifactor over correlated factor models in general). The standardised item loadings for the bifactor model are presented in Fig. 1. All items loaded strongly onto the general factor, except for one of the reverse-scored items. The ECV and OmegaH for the bifactor model was 0.763 and 0.877, respectively, providing strong evidence of ‘essential’ unidimensionality.

**Table 4:** Fit statistics for confirmatory factor analyses on reduced (18) item set

	Three-factor correlated model	Bifactor model
*χ* ^2^	517.3 *p* < .001	391.5 *p* < .001
RMSEA	0.070 [0.064–0.076]	0.063 [0.056–0.070]
CFI	0.982	0.987
TLI	0.979	0.983
WRMR	1.197	0.903
*n*	596	596

Estimation method: WLSMV

### Criterion-related validity

The 18-item PERCCI scale ranged between 0 and 54, with a mean of 39.0 and standard deviation of 10.7. There were minimal ceiling/floor effects; however, the distribution was left-skewed with 25% of respondents lying within seven points of the top of the scale. The PERCCI had moderate-to-strong correlation with both the satisfaction question (Rho = 0.700, *p* < .001) and Friends and Family Test (Rho = 0.642, *p* < .001).

An exploratory OLS regression (Table 5) also identified several trends of interest. Younger service users (aged 65–69) reported significantly higher PERCCI scores than older service users, whilst lower scores were reported by those with a dementia diagnosis (relative to respondents with functional disorders) and those referred in the 6 months preceding the survey. In relation to service receipt, those reporting seeing a registered practitioner from the CMHT reported more person-centred support than those not reporting any recent care visits. Importantly, person-centredness was *further* enhanced where care *also* included support worker visits. There was also some indication that receipt of homecare was associated with poorer perceptions of person-centredness, in line with expectations, but this result did not reach the *p* < .05 threshold.

**Table 5:** OLS regression of PERCCI

	Coeff.	Robust s.e.	*t*	*p*
Aged under 70	3.899	1.118	3.49	.001
Dementia diagnosis/other organic	− 3.502	1.263	− 2.77	.006
Referred < 6 months ago	− 5.905	1.714	− 3.45	.001
Sees registered practitioner	8.670	2.157	4.02	< .001
Sees registered practitioner * Sees support worker^1^	2.782	1.116	2.49	.013
Receives homecare	− 2.074	1.200	− 1.73	.085
Attends daycentre	2.093	1.131	1.85	.065
Constant	35.611	2.319	15.36	< .001

*n* = 410, *R*^2^ = .186, Adj *R*^2^ = .170. Shapiro–Wilk *z* = 5.721 (*p* < .001); RESET test *F*(3,395) = 1.95, *p* > .05

*Indicates an interaction effect

^1^Too few reported seeing support workers only to estimate a separate effect

### Test–retest reliability

**Fig. 2:**
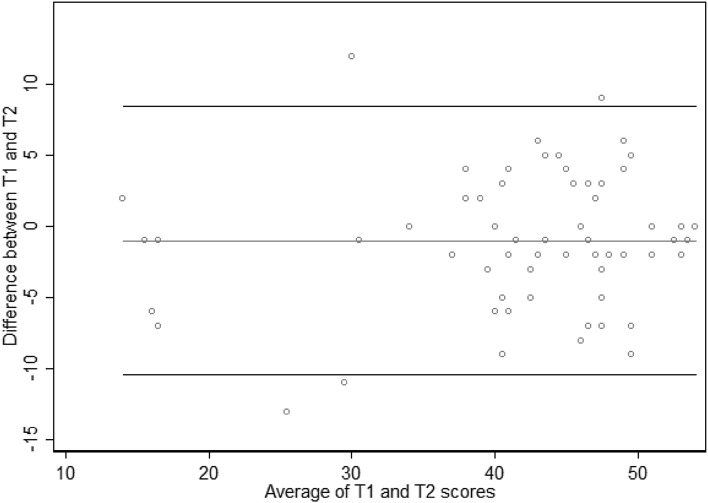
Bland–Altman plot

Test–retest reliability was assessed for 77 people completing all items at both T1 and T2. The mean time elapsed to T2 was 3.7 weeks. The ICC value of the PERCCI was estimated to be 0.886 [95% CI 0.818–0.929]. The Bland–Altman limits of agreement were − 10.44 to 8.44, as shown in Fig. 2. Three sensitivity tests were undertaken. The first restricted the analysis to those with a time elapsed to T2 of < 1 month, resulting in a trivial 0.002 improvement in the ICC. The second restricted the analysis to those with at most a one category change in the Friends and Family Test between T1 and T2 (a proxy indicating stable quality), which led to a 0.011 improvement in the ICC. Finally, mean imputation was used to complete 24 schedules with missing items, causing the ICC to reduce to 0.831 [0.759–0.883].

### Implications of removing negative items

The above analytical procedures were repeated but excluding the three negative items. A bifactor model for 15 items with only two specific factors had reasonable model fit (RMSEA = 0.073; CFI = 0.988; WRMR = 0.889). The path diagram for the fitted model is available as supplementary material. The ECV increased marginally (= 0.794), whilst the OmegaH was almost identical (= 0.879) to the 18 item version. The correlation coefficients with the satisfaction and friends and family test question, as well as the central ICC estimate, remained within ± 0.01 of the original calculations from the 18 item PERCCI.

## Discussion

Academic interest in the scientific evaluation of person-centredness has struggled with its inadequate and insufficient measurement. Without high-quality instruments, researchers have turned to inappropriate proxy measures that risk inaccurate findings. This paper presents a large-scale study of the development and preliminary psychometric testing of the new 18-item PERCCI. These results are encouraging.

### Content validity

The content validity of the PERCCI was supported by a robust approach to development and pre-testing. The 18 items were formed from primary research with older people and how they articulate care experiences, with a strong link to person-centredness assured by mapping items to a concept synthesis designed for this purpose [2]. The components of person-centredness were well represented in the 18 items, including how well practitioners understood participants’ experience of illness; their understanding of the different dimensions of their life needing support; how care was tailored to participants’ needs; whether interactions were respectful and reciprocal; and continuity in care, amongst others.

Although the items may appear applicable to all adult age groups, the argument supporting the need for a specific instrument for older age groups is supported by closer attention to item wording. For instance, items relating to involvement in decision-making might be viewed as passive, especially in the current policy climate advocating that people should be encouraged to be more directive in care decisions. PERCCI items thus evaluate whether respondents have “had a say in decisions”; whether their “opinions about care and support are respected”; and whether practitioners appear “interested in their views”. A contrast can be made with other related questionnaires predominantly developed with younger adults using more active language, such as one evaluating: “being in control” “staying independent”, “arranging support”, “choosing [options]” and similar phrases [39]. The language used in the PERCCI is entirely consistent with person-centredness, but reflects a wish for inclusion in decisions and for their contribution being valued, without expressing a desire to be responsible for control and execution of choices. This echoes wider evidence on the preferences of older adults with long-term care needs [40].

### Dimensionality and negative items

The analysis presented a robust approach to dimensionality. The initial EFA of 30 items from nearly 600 respondents identified four factors with eigenvalues exceeding 1.0, with three retained from a parallel analysis (accounting for over-factorising common in ordinal item analysis). Two factors captured person-centredness in interpersonal relationships and organisational aspects of care; and a third indicating reverse-scored items. The latter is perhaps unhelpful, since few research situations would call for an evaluation of experience in such terms. Negative items often cause difficulty in scale development, and a cottage industry has emerged exploring appropriate means of accounting for ‘method effects’. In this study, the 18 item version used three negative items. These were conceptually valuable, and in one instance (‘seeing too many different staff’) pertained to an otherwise missing aspect of person-centredness whereby service users lack continuity in care workers. By retaining the negative framing of these items, it also allowed them to remain ‘true’ to the older people’s voices that provided the statements underpinning these items. To reverse the polarity by rephrasing them positively would have risked losing the meaning or nuance intended [27]. Administratively, it is also thought that mixed positive and negative items also helps to focus respondent attention to the wording of the questionnaire. Such cognitive “speed-bumps” [41] help to avoid satisficing that artificially inflates reliability.

However, only three negatively phrased questions were retained in the final 18-item scale and it may be that they are not sufficiently valuable. Therefore, a 15-item version was examined in this paper, with equally satisfactory psychometric properties. Further work with service users could help determine whether these items were sufficiently important to keep, or whether they could be removed without undermining the content of the PERCCI overall.

### Viability of a summary score

The EFA also identified a notably dominant first factor eigenvalue, and a strong correlation with subsequent factors, signalling that a summary score spanning all items could be viable. For most research purposes, it is likely that a single metric of person-centredness would have advantages for reliability and simplicity over separate, shorter subscales. However, the construction of single summary scales from multi-factor items requires caution, since violating the assumption of unidimensionality underpinning measurement theory risks introducing bias [19, 42]. However, there are principled and falsifiable means for achieving and testing the appropriateness of such a step. The bifactor model, in this instance, evaluates the appropriateness of a ‘common factor’ interpretation of multidimensional data [34]. Here, the bifactor results provided evidence of good fit, and both ECV and omegaH statistics supplied robust evidence for ‘essential unidimensionality’ [34, 43] in that the common factor accounted for a sufficiently large proportion of variance to justify the aggregation of item scores.

The potential value of the PERCCI is highlighted by associations identified in exploratory regression. As expected, PERCCI scores were notably higher where mental health support was provided by *both* professionals and mental health support workers (rather than professionals alone). This result is supported by evidence that non-registered mental health practitioners appear to enjoy authentic relationships with service users, and have a flexible role amenable to person-centredness [37]. That domiciliary care was linked (albeit weakly) to poorer PERCCI scores was anticipated because prior research has expressed concern that personal care services in England, commissioned under austere and tightly specified local authority contracts, are incompatible with person-centredness [44–46]. Other results of interest include the low PERCCI scores amongst those recently referred to the service, perhaps indicating that care relationships and shared understandings between practitioners and service users can take time to develop.

A test–retest reliability inspection is essential for determining a measure’s quality. The ICC from 77 responders completing all 18 items at both time periods was 0.89, with a lower confidence interval bound of 0.82. This compares very well with established thresholds. For instance, an expert consensus has recommended reliability in the range of 0.70–0.80 [47], whilst a review described reliability estimates above 0.85 as ‘excellent’ [48].

## Limitations

The preliminary measurement properties reported here are encouraging but must be set in the context of the study limitations. First, the response rate was somewhat low (< 30%), raising questions over generalisability. Reasons for low completion are speculative, since cognitive pre-testing appeared to indicate high acceptability amongst older service users. Nevertheless, the achieved sample still achieved a spread of older groups often absent from scale development research, including people with cognitive impairment. Furthermore, the response rate was similar to that achieved by other surveys of this population, such as the 28% response to the NHS community mental health care survey in 2016 [49, 50]. Second, the questionnaire was developed and tested in a population restricted to older service users with mental health and social care needs living in the community, and it cannot be assumed to be of equal validity with other populations. When applying measures, it is all too common for researchers to neglect consideration of whether it has validity in the specific study population [19]. Third, content validity is established through the authors’ mapping of items to a framework of person-centredness, with no external appraisal, as yet, of this judgement. A Delphi panel or other consensus-based exercise could ascertain whether the items are accepted by other experts in person-centredness research. Finally, the study was not designed to establish the sensitivity of the instrument, nor whether it is capable of detecting ‘minimally important change’ [49]. Specifically, what is the smallest difference in PERCCI scores that service users would recognise as representing a valuable change in person-centred qualities?

Future work could also usefully examine the properties of the PERCCI under a Rasch framework. Rasch models have a particular advantage of ensuring interval-level scales by using hierarchical response patterns (akin to a probabilistic form of a Guttman pattern) to estimate the latent trait under measurement based on the ‘difficulty’ of the items affirmed/not affirmed [51]. Other advantages include a thorough examination of Differential Item Functioning, to ensure that the likelihood of a given response is not dependent on personal characteristics.

## Conclusion

The new 18-item PERCCI has promising measurement properties. Content validity is supported by sourcing its items directly from the voices of older users of community mental health and social care services, whilst mapping these to a literature-based conceptual framework of person-centredness. The 18 items have a sufficiently unidimensional factor structure, but distinct subscales can be formed for researchers with particular interest in interpersonal and/or organisation aspects of person-centredness. Its research potential is encouraging, as demonstrated in correlation and regression analysis, which broadly affirmed prior expectations. Its test–retest reliability appears to be excellent. A 15-item version, without negatively phrased items, also performed well. Further research should concentrate on validating these properties in a new sample and establishing what is the minimal change in the PERCCI score that corresponds to service users’ interpretation of a meaningful difference.
